# Deep negative volume segmentation

**DOI:** 10.1038/s41598-021-95526-1

**Published:** 2021-08-11

**Authors:** Kristina Belikova, Oleg Y. Rogov, Aleksandr Rybakov, Maxim V. Maslov, Dmitry V. Dylov

**Affiliations:** 1 Skolkovo Institute of Science and Technology, Bolshoy Blvd., 30/1, Moscow, Russia 121205; 2 First Pavlov State Medical University of St. Petersburg, L’va Tolstogo Str., 6-8, St. Petersburg, Russia 197022

**Keywords:** Digital radiography in dentistry, Applied mathematics, Computer science, Computed tomography, Three-dimensional imaging

## Abstract

Clinical examination of three-dimensional image data of compound anatomical objects, such as complex joints, remains a tedious process, demanding the time and the expertise of physicians. For instance, automation of the segmentation task of the TMJ (temporomandibular joint) has been hindered by its compound three-dimensional shape, multiple overlaid textures, an abundance of surrounding irregularities in the skull, and a virtually omnidirectional range of the jaw’s motion—all of which extend the manual annotation process to more than an hour per patient. To address the challenge, we invent a new workflow for the 3D segmentation task: namely, we propose to segment empty spaces between all the tissues surrounding the object—the so-called negative volume segmentation. Our approach is an end-to-end pipeline that comprises a V-Net for bone segmentation, a 3D volume construction by inflation of the reconstructed bone head in all directions along the normal vector to its mesh faces. Eventually confined within the skull bones, the inflated surface occupies the entire “negative” space in the joint, effectively providing a geometrical/topological metric of the joint’s health. We validate the idea on the CT scans in a 50-patient dataset, annotated by experts in maxillofacial medicine, quantitatively compare the asymmetry given the left and the right negative volumes, and automate the entire framework for clinical adoption.

## Introduction

Our study began from the following simple question while we were performing a very tedious manual annotation of a compound three-dimensional (3D) structure. **Q**: Instead of finding the exact contours that circumscribe the 3D object, can we segment *the air* that fills the gaps within its parts? What deep neural network architecture would accomplish that, given the gaps are the *absolute complements* to the annotation labels? To find answers, we geared up with the most complex 3D object we could find.

Some of the most structurally complex objects in the human body are indisputably the joints, in general, and the *temporomandibular joint* (TMJ), in particular. TMJ is a bilateral joint formed by the *mandibular* and the *temporal* bones of the skull, differing from the other joints anatomically and functionally^1,2^. TMJs enable functions like chewing and speaking. Several medical research groups still actively debate trying to explain the kinetic function of the TMJ joint, its multiple degrees of freedom, and even its relation to a plethora of known illnesses (maxillofacial ones and beyond^2,3^). Accurate interpretation of TMJ images has become essential in a variety of clinical practices, ranging from the basic assessment of wear and tear (e.g., osteoarthritis) to intricate surgical interventions (e.g., arthroplasty). The lack of trustworthy automation of the basic diagnosis-assisting routines (such as tendon segmentation or a measurement of the cartilage wear) stems from the fact that such compound joints have extremely intricate 3D anatomy and a variety of surrounding tissues of perplexed morphologies and textures^4^. We show a number of 3D examples of the TMJ’s complex geometries in the supplementary material.

Millions of people suffer from temporomandibular disorders (TMDs), having such symptoms as a limitation or a deviation of the range of the jaw’s motion, certain TMJ sounds, associated headache, and the very pain in the joints. Orthodontic, maxillofacial, and plastic surgeries point to the other large related cohort of patients. Despite being that common, the diagnostics of all of the mentioned TMJ symptoms remains very challenging^5^, and the current clinical practice entails very rudimentary *linear or 2D* measurements of the joint’s tissues. Such measurements have obvious shortcomings: they are subjective, time-consuming, and not accurate enough due to the in-plain estimations. In fact, significant outcome differences were reported when TMJ is measured in 2D *vs.* in 3D^6^. True 3D characterization of TMJ in medical images is essential for improving various clinical practices, including dentistry, orthodontics, maxillofacial and plastic surgeries.

Manual 3D annotation of the TMJ is usually undertaken only by the top hospitals, requiring expertise of the maxillofacial doctors, that of a 3D modelling technician, and a long collaborative effort to draw a fitting 3D model of the jaw and of the other head parts involved^7^. In fact, there is simply *no standardized annotation workflow* for contouring the TMJ structures *even manually* today. This manuscript proposes a new protocol for such an annotation and proposes a method for its end-to-end automation in clinical use.

### Medical background

#### Joint health assessment

Joint health assessment is essential in many clinical practices, ranging from basic orthopedics to complex maxillofacial and plastic surgeries^8–10^. While different metrics of the health of the inter-articular space have been proposed, the exact definition of the joint space boundaries is still a matter of debate (see, *e.g.*, wrist^11^, knee^12^, or hip^13^). Conventionally, the diagnosticians resort to basic in-plain measurements of the linear dimensions between some anatomic reference points in the radiological scans to assess the health of the joint^6^. Several recently proposed automation techniques^14–16^ demonstrated robustness and reproducibility required for expanding the assessment to 3D, still confirming the disagreement in the definition of the joint space volume of interest, which could be attributed to the vague borders between the soft and the connecting tissues as well as their intricate texture and anatomic structure^17^. The current practices indicate the need for a robust and repeatable joint space assessment method that would operate both volumetrically and automatically.

#### TMJ space specifics

For TMJ space, this demand is especially well-articulated, because the proper joint space is required for the normal free movement of the jaw (or the *mandibular condyle*) and the movement of the articular disc within the joint. The widening or narrowing of the joint space may point to some type of TMJ pathology, whereas the difference between the left- and the right-side joint spaces is the main cause of facial asymmetry, even if the bones themselves remain symmetrical^5^. Moreover, the development of the TMJ space is highly individualized, making a comparison between the patients difficult^18^. Another unanswered question in the TMJ community is the definition of the “ideal” mandibular condyle position, stimulating the debates between gnatologists and orthodontists and affecting the development of a single joint health assessment standard^19^. Thus, the high variability across different patient cohorts^4^, the lack of agreement on the joint’s ‘home’ position, and the lack of a proper joint space assessment standard, hinder the application of modern data-dependent deep learning tools to address the challenge.

#### Current clinical TMJ space assessment standards and metrics

Because of the complexity of TMJ, the 2D slice-by-slice visualization is insufficient for finding the cause of a given symptom, requiring a true 3D reconstruction to describe its anatomy. Yet, many doctors have to resort to rudimentary linear measurements of the objects in the 2D scans. Among the currently used metrics for TMJ examinations are the horizontal condylar angle (HCA), sagittal ramus angle (SRA), medial joint space (MJS), lateral joint space (LJS), superior joint space (SJS), anterior joint space (AJS), and the width/depth of mandibular fossa (FW, FD)^20^. Being selected by the eye and being based on imprecise reference points, these metrics can only depict the 2D representation of the 3D pattern. In our work, we suggest to consider the comprehensive volumetric measures instead, such as the volume and the surface area of the joint space, proposing the most complete morphological and topological description of the TMJ.

### Technical background

#### Object localization on medical scans

Automatic localization of objects of interest is a prerequisite for many medical imaging tasks, as it can narrow down the field of view to the important structures. As of today, there are several approaches for detecting specific areas of various shapes and sizes such as body parts, bone tissues, organs, nodules, and tumors in 3D MRI and CT images^21–26^. Completely autonomous cropping in medical images has been reported^21^. It is a common practice to use a cascaded approach, consisted of several steps: object localization and object segmentation or another required action. The first step is to localize the object from the entire 3D scan, and then provide a reliable bounding box for the more refined steps^27^, Mask R-CNN^28^, 3D RoI-aware U-Net^23^, segmentation-by-detection^13^, etc.).

#### Medical image segmentation

With the advent of artificial intelligence to medical image computing, a wide range of image segmentation challenges were successfully tackled by deep learning methods (see Refs.^29–32^ for review). In particular, significant advances were made by the architectures based on the Convolutional Neural Networks (U-Net^33,34^, V-Net^35^, U-Net++^36^, MD U-Net^37^, Stack U-Net^38^, etc.). Among many anatomical objects that have been drawn to the focus of the segmentation challenges, the human bones have remained the subject of active research^39,40^. Modern high-resolution imaging^41^ and the segmentation approaches enabled thorough quantitative studies which nowadays help assess changes in the bone structure^42^ and porosity^43^.

Of specific value to our task, are the 3D U-Net^34^ and the attention-gated 3D U-Net^44^ architectures that take advantage of efficient GPU computing, the ability to achieve high precision with a fewer training samples, and the capability of “learning where to look” with the class-specific pooling^45^. To automate the negative volume segmentation task, we first needed to segment the major bones (mandibular and temporal bones), which eventually draw us to select the V-Net architecture^35^. V-Net is similar to 3D U-Net but is more prone to convergence thanks to learning the residual function along the way. The summary of the architecture selection is covered in “Mandibular condyle and temporal bone segmentation” section. Once the bone segmentation was automated, we proceeded with the segmentation of the space between the bones. For that, we introduced a new *inflation* procedure that gradually fills the space between the inner structures of the joint until the entire negative volume is occupied. The inflation procedure and the full segmentation pipeline are described in “Automatic pipeline: segmentation of negative volume” section.

#### Mesh inflation

Deformation, inflation or deflation are commonly employed in complex 3D reconstruction problems to boost the model quality by detailing the meshes. Modern physics-based mesh deformation and generation methods, combine robust constraint optimization and efficient re-meshing^46^, which proved useful in medical imaging^47,48^ but still requires additional evaluation of the nesting feasibility criteria, often viewed as constraint optimization problems for meshes^49^.

### Contributions

The key contributions of our paper are the following:

- New paradigm for segmentation of the ‘air gaps’ within complex 3D objects (the concept of “Negative Volume”) using a deep neural network.
- New *manual* annotation workflow for negative volume segmentation in the human joints. It is multiple orders of magnitude more descriptive than current clinical standard.
- First *automatic* end-to-end pipeline for extraction of negative volumes within a human’s joint, incorporating deep learning-based localization, segmentation, and surface mesh inflation.
- New volumetric measure of a joint’s health based on its symmetry properties via the state-of-the-art topological cloud-to-cloud metrics.

In this work, we propose a new workflow, by suggesting to shift the focus from the segmentation of the hard-to-contour anatomical structures within the joint to the segmentation of the spaces between these structures (the gaps). We have called the method “negative volume” reconstruction and presented a new method of manually annotating such a volume in “Manual annotation pipeline: negative volume concept” section. Also, we present an end-to-end pipeline for extracting deep negative volumes from the CT scans to automate and to improve the manual one. Our fully-automatic 3D deep negative volume segmentation/reconstruction approach is described in “Automatic pipeline: segmentation of negative volume” section.

## Methods

This section covers the concept and the workflow to generate negative volumes via two pipelines: manual 3D annotation (“Manual annotation pipeline: negative volume concept” section) and an end-to-end automatic approach which is even more descriptive than the proposed manual one (“Automatic pipeline: segmentation of negative volume” section), suggesting a new metric for assessing the health of joints.

### Manual annotation pipeline: negative volume concept

To reveal the concept of negative volume, we introduce a new method for examination of complex joints that takes advantage of all available 3D information acquired by an imaging modality.

**Figure 1:**
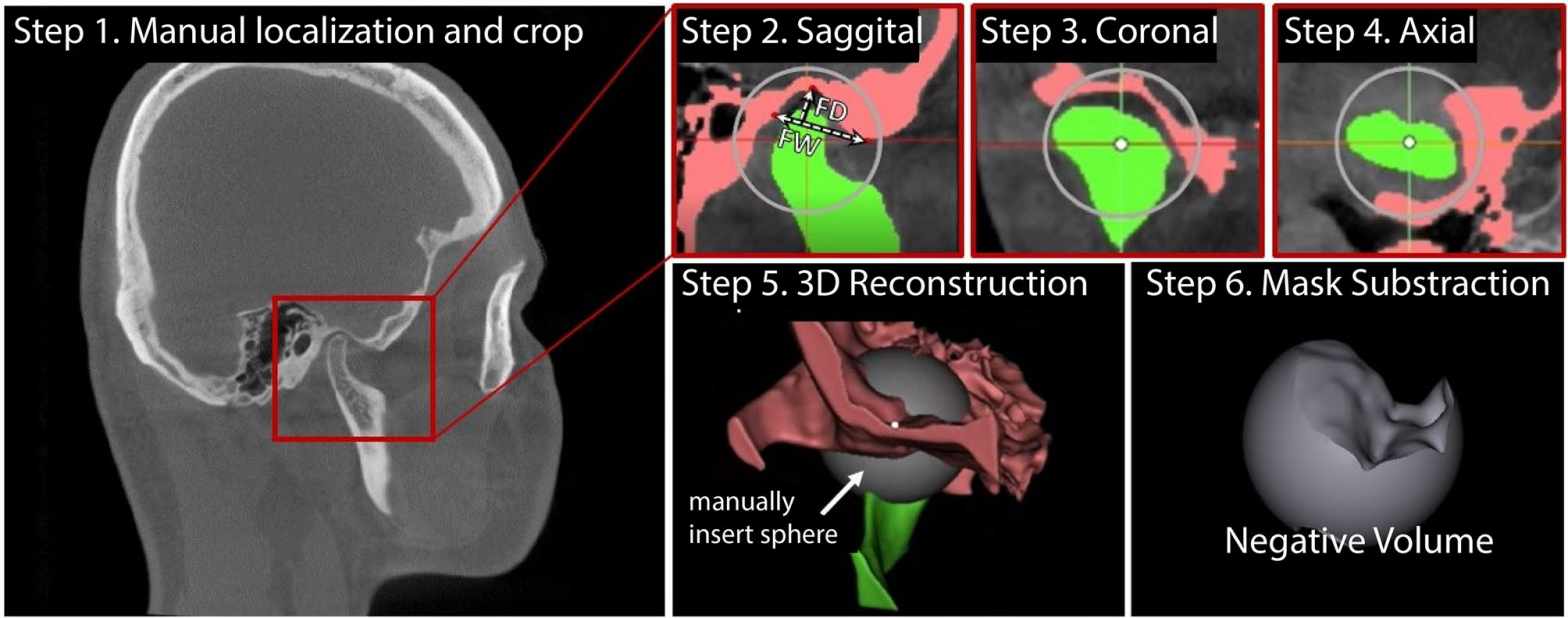
Proposed steps for manual negative volume annotation in TMJ (left to right). The process requires drawing masks around complex structures of mandibular condyle (green) and temporal bones (red) in all three views (saggital, coronal, and axial) for each slice of the volume of interest (VOI), until the resulting 3D reconstruction allows to subtract the negative “ball” from a manually inserted sphere. Such annotation *takes about 1 hour per patient*. Figure created with Incscape v.1.1, https://inkscape.org/.

Figure 1 proposes *volumetric characterization* of a joint, with TMJ taken as an example. The method targets extraction of the *empty space* between the various tissues surrounding the joint, which we intuitively call a “negative volume”. To extract it, the proposed manual annotation pipeline entails drawing a series of 2D masks for the mandibular condyle (MC) and for the temporal bone (TB) in a cropped sequence of the original DICOM, a resulting 3D reconstruction of the volumes of the MC and TB bones, a manual (rough) positioning of a 3D sphere within the joint center, and a consequent subtraction of the mask volumes from the sphere.

Unlike the current clinical examinations^50^, where the width and the depth of the mandibular fossa are measured (“FW” and “FD” in Fig. 1), the true volumetric “negative ball” extracted from the joint is far more informative. It takes more than an hour to annotate one patient; if automated, it could be quickly adopted in the clinical practice as a new measure of joint’s health.

### Automatic pipeline: segmentation of negative volume

**Figure 2:**
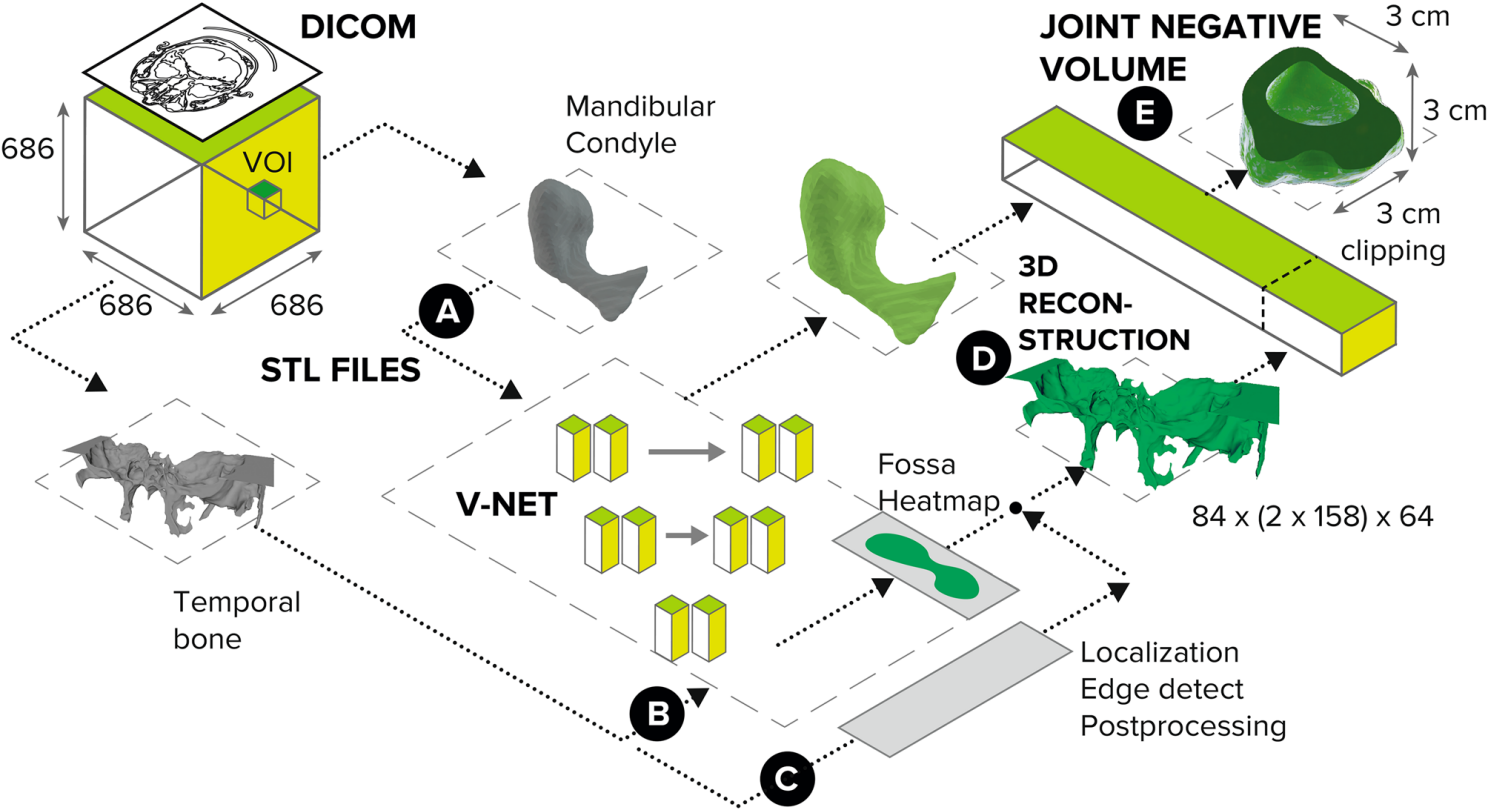
End-to-end pipeline for Deep Negative Volume Segmentation in joints. Segmentation of MC and TB are shown as step A and step B, respectively. Step C and step D represent classical image enhancement of both bone reconstructions. Fig. 3 shows “inflation/clipping” block (step E) in detail. Figure created with Incscape v.1.1, https://inkscape.org/.

We now proceed to automating an end-to-end pipeline based on the approach in Fig. 1 but with several principle differences which stem from the fact that such negative volumes are impossible to annotate in a sufficient number manually (to train a typical 3D network). The proposed pipeline consists of the following steps: data preprocessing, volume of interest (VOI) selection, segmentation of the TB and MC bones, 3D reconstruction of the segmentation results, inflation of the MC volume to fit into the mandibular fossa, and, finally, extraction of the negative volume by clipping (see Fig. 2).

#### Data preprocessing

Basic DICOM data normalization and confirmation of the co-alignment of the ground truth annotation masks are done as the first step. The data preprocessing consisted of min-max normalization of DICOM data and voxelization of Standard Triangle Language (STL) models. Details of STL models voxelization and further data augmentation are given in “Experiments” section.

#### VOI selection

We have approached the localization of TMJ VOI bounding the bones (MC and TB) as a segmentation problem at a lower resolution, based on the available memory and size of input data. To perform localization of joint we utilize V-Net model, which has proven itself as an accurate enough voxel-based model with fast convergence. For our case, we resize the raw images to a lower resolution $160\times 160 \times 160$ using bicubic interpolation to preserve available memory. This step results in two cropped volumes of various sizes to be used for training the segmentation neural network: both the left and the right joints with separate masks for MC and TB.

#### 3D bone segmentation: (A) MC and (B) TB bones

One has to resort to architectures for 3D segmentation due to the complex structure and texture of the bones in that part of the skull (especially, the TB which has many irregularities). The V-Net architecture proved to work best for the MC, as well as for the complex TB bone. Full comparison of the architectures is given in Table 2, with V-Net being better for deployment due to its faster convergence (to segment both MC and TB).

#### (C) Classical image enhancement

While MC segmentation via V-Net proved satisfactory (step A in Fig. 2), the TB segmentation (step B in Fig. 2) needed to be enhanced by passing the original data through a classical processing route (step C in Fig. 2): namely, we applied the removal of noise, closing edges, morphological smoothing (such as erosion and dilation), and 3D Canny edge detection filters. The sequence of these operations is completely automated and the result is fused with the *fossa* heatmap, generated by V-Net, to provide a single TB mask. Notably, the step C could be removed altogether once a sufficiently large number of manual annotations of the TB is collected. Or, it can be viewed as ”compensation” for the complex irregularities encountered in the joint, which would otherwise require a lot of annotation for training.

#### (D) 3D reconstruction

To reconstruct 3D models, 116 equidistant consecutive sections with a pitch of 0.4 mm and a bounding box dimension of 103 px $\times$ 158 px were used. The fused surfaces of the interfaces between the articular disk, MC, and TB were subjected to median averaging, 2D-filtering, and interpolation, entailing filter radius matching (to fit the size of irregularities) and the edge detection applied in a slice-by-slice manner.

#### (E) Negative volume inflation

**Figure 3:**
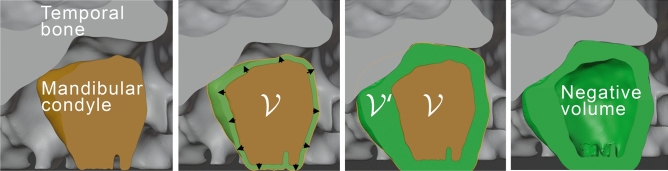
Proposed negative volume *inflation routine* seen in TMJ cross-section (frontal view): (1) segmented MC bone is a starting point (mesh ${\mathcal {V}}$ ), (2) surface of MC spreads along the normals, (3) inflated MC reaches bounding volume defined by TB model (mesh ${\mathcal {V}}^{\prime }$), (4) MC removal and clipping of the neck of the mandible generates the negative volume. Figure created with Incscape v.1.1, https://inkscape.org/.

Figure 3 summarizes how the mesh ${\mathcal {V}}$ of the reconstructed MC bone is inflated along the normals to maximize similarity with the *fossa* space surrounding the TB mesh ${\mathcal {V}}^{\prime }$. Inflating the mesh ${\mathcal {V}}$ belongs to a class of optimization problems that are accompanied by the Laplacian regularization to ensure a smoother shape^51^. Boolean difference of the two meshes ${\mathcal {V}}^{\prime }\setminus {\mathcal {V}}$ provides the final negative volume of interest.

#### Symmetry metrics

Having received both the left (${\mathcal {L}}$) and the right (${\mathcal {R}}$) negative volumes, the doctors can proceed to any accurate volumetric measurements, relevant to a given set of particular symptoms and conditions at hand. In maxillofacial practice, for instance, it is quite common to estimate the ${\mathcal {L}}{-}{\mathcal {R}}$ symmetry^5^ of the TMJs, which directly correlates with the jaw’s alignment. For that, we suggest to use a volumetric measure based on the Hausdorff cloud-to-cloud distance. To estimate the symmetry between the two negative volumes, we define the Hausdorff distance for two point sets (${\mathcal {L}}$ and ${\mathcal {R}}$) on a metric space $({\mathbb {R}}^3,d)$, where *d*(*l*, *r*) is the Euclidean distance between the points *l* and *r*. The Hausdorff measure is a well-known and a robust metric that exists in many programming libraries. Many other possible metrics could be also proposed though, e.g., $S_{LR}$ the ratio of the mesh surface areas of both negative volumes $S_L$ and $S_R$, where the lower index corresponds to the left and right volume respectively.

1 $$\begin{aligned} H_{LR}= & {} \max \left\{ \sup _{{l} \in {\mathcal {L}}} \inf _{r \in {\mathcal {R}}} d(l, r), \sup _{r \in {\mathcal {R}}} \inf _{l \in {\mathcal {L}}} d(l, r)\right\} , \end{aligned}$$

2 $$\begin{aligned} S_{LR}= & {} \frac{\max \left\{ S_L, S_R\right\} }{\min \left\{ S_L, S_R\right\} } \end{aligned}$$

We report measurements with both proposed symmetry metrics in the Results Section. These metrics are as descriptive as possible and ought to replace the simplistic conventional linear measurements.

### Inflation versus 3D segmentation: Why choose inflation?

Supervised 3D segmentation models typically require extra labels to perform well. Given the time required to annotate our negative volumes manually ($\sim$1 h, see Fig. 1), one would have to go through a very long annotation process to generate a proper dataset. Instead, we use lighter models for well-discernible bones and perform 3D inflation of the mesh, effectively mitigating the shortage of the labels and—importantly—also preserving the interpretability because the inflated volumes naturally ‘occupy’ the available empty space in the joints.

**Figure 4:**
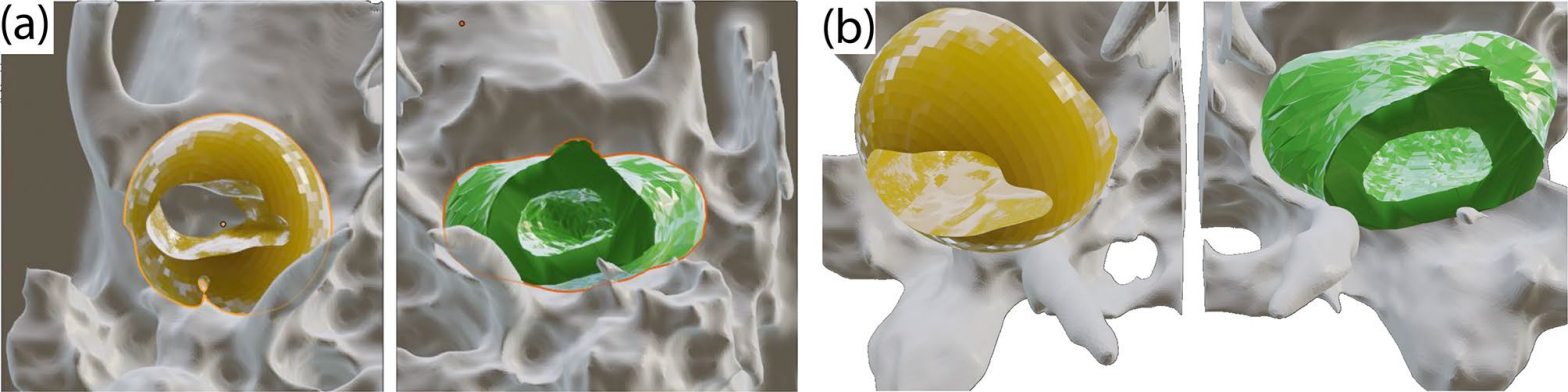
Proposed manually annotated (yellow) versus machine-generated (green) negative volumes. Rendered regions of the TB are shown in gray. Views: (**a**) axial, from bottom (**b**) same, tilted. Figure created with Incscape v.1.1, https://inkscape.org/.

Table 1 summarizes the key differences between the manual approach and the proposed automatic pipeline. Although our manual approach has a number of advantages over the clinical joint assessment methods, the machine-generated negative volumes are even better, being faster and entailing a more informative outer surface of the volume (see examples in Fig. 4 and in the supplement).

**Table 1:** Summary of key characteristics between clinical and proposed methods.

Feature	Clinical standard	Proposed manual NV	Proposed automated NV
Allows 3D measurements	–	+	+
Number of extracted parameters	$\sim$2–16	$\sim$1–2$\times 10^3$	$\sim$2–3$\times 10^3$
Defines exact anatomical shape	–	–	+
Resilient to re-positioning	–	–	+
Hands-free report/automation	–	–	+
Segmentation time	0.5 h	1 h	4 s

NV stands for the negative volume.

## Experiments

### Dataset

To validate our deep negative volume segmentation approach, we use a local dataset containing high-resolution DICOM scans of the heads of 50 patients. The dataset was acquired at “Clinica na Griboyedova” dental clinic (Saint-Petersburg, Russia) specially for conducting this research. The dataset acquisition and the retrospective study were carried out in accordance with relevant guidelines and regulations. The experimental protocols were approved by a named institutional committee at Pavlov First St. Petersburg State Medical University. All patients involved in the study were adults (>18 y.o.) and signed an informed consent permitting the use of their data in anonymized format. We note that there are no publicly available datasets suitable for this study because of the sensitive biometric data contained within the head CT scans (e.g., face and teeth).

All acquired head CT scans have the resolution of 0.4 mm and the dimensions of 686 \times 686 \times 686 pixels. The ground truth masks [20 STL models of 10 patient’s mandibular heads (*i.e.*, left and right TMJs)] were obtained after the manual annotation by two experienced orthodontists following the pipeline shown in Fig. 1 in the MIMICS software^52^. The STL models were voxelized by the subdividing method: a mesh was scaled down until every edge was shorter than the spatial resolution.

The train-test split was done by patient id, as it is a standard for medical datasets. All models were trained using 5-fold cross-validation on 10 patients with annotated masks. This was made to have all available labeled data in the training group, thus, increasing the accuracy for the remaining 40 patients in the hold-out test. To further minimize the overfitting problem originating from the limited training set, we applied a large variety of data augmentation techniques: random 3D rotation, horizontal flipping, contrast, translation, and elastic deformations. All the augmentation techniques were applied on the fly during training.

### Training of the neural network

#### Implementation details

The deep learning pipeline is implemented using Pytorch framework^53^. Experiments were conducted on a server running Ubuntu 16.04 (32 GB RAM); the training was done on NVIDIA GeForce Ti 1080 GPU (11 GB RAM). In all experiments, we use a 5-fold cross-validation and report the mean performance. The volume inflation routine was implemented using the Blender Python public API^54^. The segmentation computational costs estimations are 322.5 GFLOPs for V-Net *vs.* 840.5 GFLOPs for 3D U-Net, and the inflated 3D volume can be computed in $\mathcal {O}(n^2)$ FLOPs^55^.

#### TMJ localization

For localization training, $160\times 160\times 160$ images and a combination of both masks (TB and MC) are used with a batch size of 1 for memory considerations. We use Adam optimizer with learning rate 0.001 and parameters $\beta _1 = 0.9$, $\beta _2 = 0.99$. The weight decay regularization parameter is equal 0.01. Linear combination of Cross-Entropy (CE) and Dice loss was used as a loss function to optimize both a pixel-wise and overall quality of segmentation. After obtaining a rough segmentation of the joint area, automatic postprocessing was performed, including thresholding based on the minimum method and morphological operations to remove outliers.

#### MC and TB segmentation

The segmentation models are trained on $112\times 144\times 64$ patches form resulted VOIs, which differ slightly on all scans. Adam optimizer is used with initial learning rate of 0.0001. Each model is trained for 100 epochs (8000 iterations) to ensure convergence. We did not perform specific hyperparameter tuning and used fixed hyperparameters for an honest comparison. We run the training with Cross-Entropy (CE), Dice loss (D), or their linear combination to evaluate the impact of these metrics on segmentation performance. Dice score (DICE), Cross-Entropy, and Hausdorff distance (HD) were used to evaluate the performance of segmentation.

## Results

### Joint localization

The V-Net model used for localization task reached the Dice coefficient $64.6 \pm 0.3 \%$ and Cross-Entropy $0.040 \pm 0.001$ for evaluation of coarse segmentation on full CT scans and MSE is $7.940 \pm 2.009$ for determination of bounding boxes around joints. We show the visual results of localization together with the resulting VOI boundary in the supplementary material. It confirms that the achieved quality is sufficient to approximate the location of the joint, because in the collected dataset, as well as in general clinical practice, there is no single way to determine the exact boundaries of the joint.

### Mandibular condyle and temporal bone segmentation

Table 2 shows the results of the 3D U-Net, 3D U-Net with attention, and V-Net models trained with different loss functions for the bone segmentation blocks in Fig. 2. It justifies selection of V-Net architecture trained with D+CE, which perform best for segmenting MC in terms of all chosen metrics and achieves an average Dice score of 91.4 % and Cross-Entropy of 0.154, which is of the state-of-the-art level in various well-annotated segmentation reports^56,57^. For TB segmentation, V-Net also outperform 3D U-Net and 3D U-Net with attention in terms of HD and it is not much inferior in other metrics. We note that the TB annotation can be very rough due to such a complex shape of this bone, making it very hard to gauge segmentation performance by simple comparison with the ground truth labels (see the supplemental material for visual assessment and Fig. 4). The relatively high values of the Hausdorff distance in Table 2 support this notion and reinforce the idea behind the auxiliary classical processing (step C in Fig. 2) required for the insufficiently annotated datasets.

**Table 2:** Mandibular condyle (MC), temporal bone (TB) and negative volume (NV) segmentation results.

Obj.	Score	3D U-Net	3D U-Net+Att.	V-Net CE	V-Net D	V-Net D+CE
MC	DICE	91.4 ± 5.3	89.8 ± 8.2	90.9 ± 4.5	90.9 ± 6.3	91.4 ± 4.8
	CE	0.320 ± 0.003	0.320 ± 0.005	0.201 ± 0.075	0.175 ± 0.024	0.154 ± 0.053
	HD	14.7 ± 20.8	15.2 ± 21.6	11.9 ± 15.7	11.5 ± 20.1	10.5 ± 21.2
TB	DICE	75.5 ± 8.8	75.8 ± 8.4	75.9 ± 6.9	76.7 ± 6.8	76.3 ± 7.2
	CE	0.463 ± 0.043	0.462 ± 0.035	0.383 ± 0.088	0.396 ± 0.093	0.416 ± 0.100
	HD	29.8 ± 11.5	29.9 ± 11.3	27.9 ± 11.5	28.3 ± 10.7	27.6 ± 10.9
NV	DICE	78.0 ± 10.6	77.8 ± 9.6	78.1 ± 8.8	78.2 ± 8.3	77.7 ± 7.7
	CE	0.344 ± 0.016	0.349 ± 0.012	0.402 ± 0.019	0.396 ± 0.024	0.406 ± 0.022
	HD	15.8 ± 18.8	15.5 ± 17.6	19.1 ± 16.2	18.3 ± 16.9	18.7 ± 17.8

Notice that the whole-object 3D segmentation of the manually annotated “balls” from Fig. 1 need more data to work properly, justifying the development of our automated pipeline which just needs MC and TB masks.

Here *CE*, *D* are Cross-Entropy and Dice loss, respectively. *DICE* (measured in $\%$) and *HD* are Dice score and Hausdorff distance. *Att.* stands for the attention-gate architecture.

**Table 3:** Proposed negative volume symmetry metrics $S_{LR}$ and $H_{LR}$, and the rudimentary linear measurements currently used in clinics (“FW” and “FD” marked in Fig. 1).

Patient	$FW_{L}$, mm	$FD_{L}$, mm	$FW_{R}$, mm	$FD_{R}$, mm	$S_{LR}$	$H_{LR}$
1	15.6	6.8	15.2	6.7	1.02	1.79 ± 0.25
2	14.6	6.3	16.4	7.2	1.03	1.82 ± 0.28
3	17.5	7.3	16.7	7.4	1.02	1.48 ± 0.31
4	18.3	7.9	18.9	7.5	1.15	2.34 ± 0.29
5	16.6	7.7	21.2	6.8	**1.17**	**2.84** ± 0.27
6	16.3	6.8	19.8	6.7	**1.21**	**3.01** ± 0.28

Bold font indicates the unhealthy joints.

### Machine-found negative volumes

3D-reconstructed volumes of the segmented MC bones are then “inflated” as shown in Fig. 3. The result of such operation for a single patient is shown in Fig. 4, which compares the manually annotated negative “ball” (yellow, pipeline of Fig. 1) and the non-spherical machine-generated negative volume (green, pipeline of Fig. 2). Remarkably, despite being much more informative than the linear measurements, our manual annotation solution still struggles to portray the full complexity of the “negative space” in the joint. On the contrary, the machine-generated negative volumes effortlessly occupy the space available within the joint and, thus, summize *complete volumetric characterization* of the joint. Our end-to-end algorithm generates such volumes $\sim$100-fold faster than the human, taking about 4 seconds to compute.

**Figure 5:**
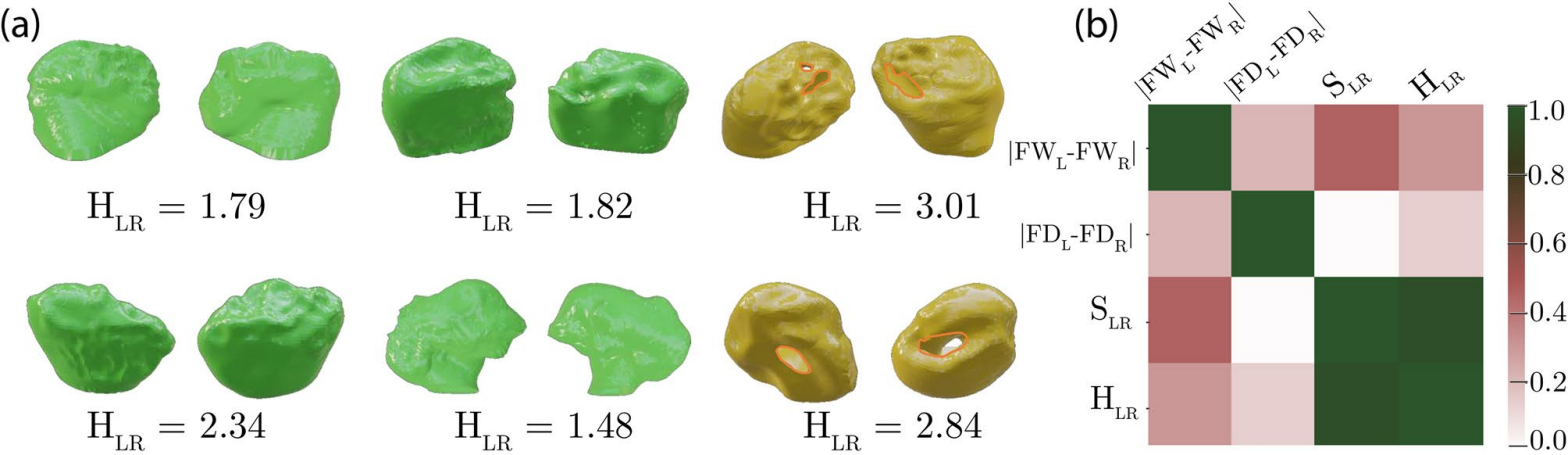
(**a**) Negative volumes of 6 patients from the Table 3 and their symmetry metrics. Notice unevenly worn out joints in the last column (TMD patients). (**b**) Correlation between the proposed and the state-of-the-art symmetry measures for the entire dataset. Heatmap is generated using Matplotlib v.3.4.2.

We generated pairs of negative volumes for all 50 patients, and showed measurements for six of them in Fig. 5 and in Table 3. Although rudimentary, the clinical measurements correlate with the proposed volumetric metrics in the task of detecting the worn joints (see 50-patient heatmap in Fig. 5b), implying that the new volumetric metrics $S_{LR}$ and $H_{LR}$ could be proposed for adoption to the current practices of the maxillofacial medicine.

## Discussion

Notice that the patients with confirmed jaw misalignment (patients no. 5 and 6 in Table 3 and Fig. 5a) have distinct pathological profiles in the negative volumes. These cases emphasize how important it is to have the full volumetric representation of the empty space within the joint. What could also be concluded from Table 3, is that the proposed metrics are not exclusive: we observe that $S_{LR}$ is more specific and is better suited for large asymmetry, whereas $H_{LR}$ is more sensitive to miniature differences in the shape, such as those in the TB bone. Modern topological metrics, e.g. Wasserstein distance, could further enhance asymmetry detection by taking advantage of the optimal transport theory^58^. Another line of future work calls for continuation of data collection and annotation. We publish the code of our end-to-end pipeline (github.com/cviaai/DEEP-NEGATIVE-VOLUME) and can envision its seamless integration into active learning tools to alleviate the annotation burden.

From the *technical standpoint*, we proposed a new intuitive hybrid strategy for medical 3D image segmentation, entailing new manual annotation pipeline, localization-based image enhancement, deep learning-based segmentation, and surface mesh inflation. The framework extracts “negative volumes” in complex anatomical structures in an end-to-end manner, which we validated on a head-CT dataset by segmenting the most complex human joint (the TMJ) together with maxillofacial experts. Our method is two orders of magnitude faster than the manual segmentation and more informative compared to the current practices because it generalizes the standard “flat” measurements to the three-dimensional case and, thus, uses all available information in the patient’s scan. The proposed 3D measure depicts topological properties of the joint space more accurately and is agnostic of the anatomic reference points or the conventions about the border of the joint, otherwise required for the manual pipelines. We, therefore, propose this method as a new joint health assessment technique for the large cohort validation and consequent clinical adoption.

From the *clinical translation standpoint*, the proposed method of visualization of TMJs could be the first step in a natural attempt to standardize volumetric measurements in intricate 3D anatomies. This instrument can perform the exact measurement of the percentage of the intact joint space tissues regardless of different protruding elements, complex shapes, textures, or the individual patient-specific variations of the joint space. The devised segmentation and reconstruction pipeline, along with its negligible computational time, can help standardize TMJ health and facial symmetry assessments in different hospitals and in different research groups, ultimately promising answers to the open questions about the “ideal” state of TMJ in the jaw’s normal position and about its complete role in the musculoskeletal health.

Future direction of this work can be focused on refinement of the proposed method. The effort should be dedicated to increasing the size of the annotated dataset, to a search for the optimal ways to reconstruct the negative volumes, and to adaptation of the proposed technique to the other human joints, such as wrist joints, knees, hips, etc.

## Conclusions

Modern computer vision software shows impressive accomplishments in extracting and understanding a plethora of 3D object shapes from various imaging applications. Following the advent of deep learning (DL), the segmentation of 3D objects could be now done with excellent quality. Yet, the segmentation of the truly intricate compound 3D objects still remains an essential challenge. We proposed an elegant and an intuitive approach to avoid the hard-to-annotate regions of a compound 3D object, and—instead—learn how to segment ‘the air’ within the 3D object of interest. We coined this ‘air’ as a “Negative Volume” and proposed the first DL framework for segmenting them automatically.

In this work, we showed 3D segmentation of a particularly complex joint in the human jaw (allegedly, the most complex one in the body). The method, however, is universal, and the methodology of deep learning-based segmentation of negative volumes could impact disciplines beyond healthcare, ranging from the additive manufacturing, to the seismic sensor 3D data, to detecting underground objects in oil and gas, to extracting complex scenes from LIDAR data in self-driving cars.

## Supplementary Information

Supplementary Information 1.

Supplementary Information 2.
