# Supplementary material for: Deep negative volume segmentation
Source: Sci Rep. 2021 Aug 11;11:16292. doi: 10.1038/s41598-021-95526-1 (PMC8357924; doi:10.1038/s41598-021-95526-1)
Supplement: Supplementary file 1 — Supplementary Information 1. [file 41598_2021_95526_MOESM1_ESM.pdf]

Deep Negative Volume Segmentation: Supplementary material

Kristina Belikova[1,*], **Oleg Y. Rogov**[1,*], **Aleksandr Rybakov**[2], **Maxim V. Maslov**[2], **and Dmitry V. Dylov**[1,**]

[1]Kristina Belikova, Oleg Y. Rogov and Dmitry V. Dylov** are with the Skolkovo Institute of Science and Technology, Bolshoy blvd., 30/1, Moscow 121205, Russia
[2]Aleksandr Rybakov and Maxim V. Maslov are with the First Pavlov State Medical University of St. Petersburg, Russian Federation, L'va Tolstogo str., 6-8, St. Petersburg, 197022, Russia.
[*]Equal contribution
[**]Corresponding author: d.dylov@skoltech.ru

1 Overview

Our Supplementary material is structured as follows. First, in Section 2, we present details about the dataset, demonstrating the anatomical diversity and shape complexity of different TMJ joints. In Section 3, we illustrate the major difference between the manually annotated negative volumes and the ones directly segmented given that 3D annotation. In Section 4 we present localization results. In Section 5, we then discuss the segmentation performance as a function of the training set size and of the training loss function, given the metrics considered in the main text.

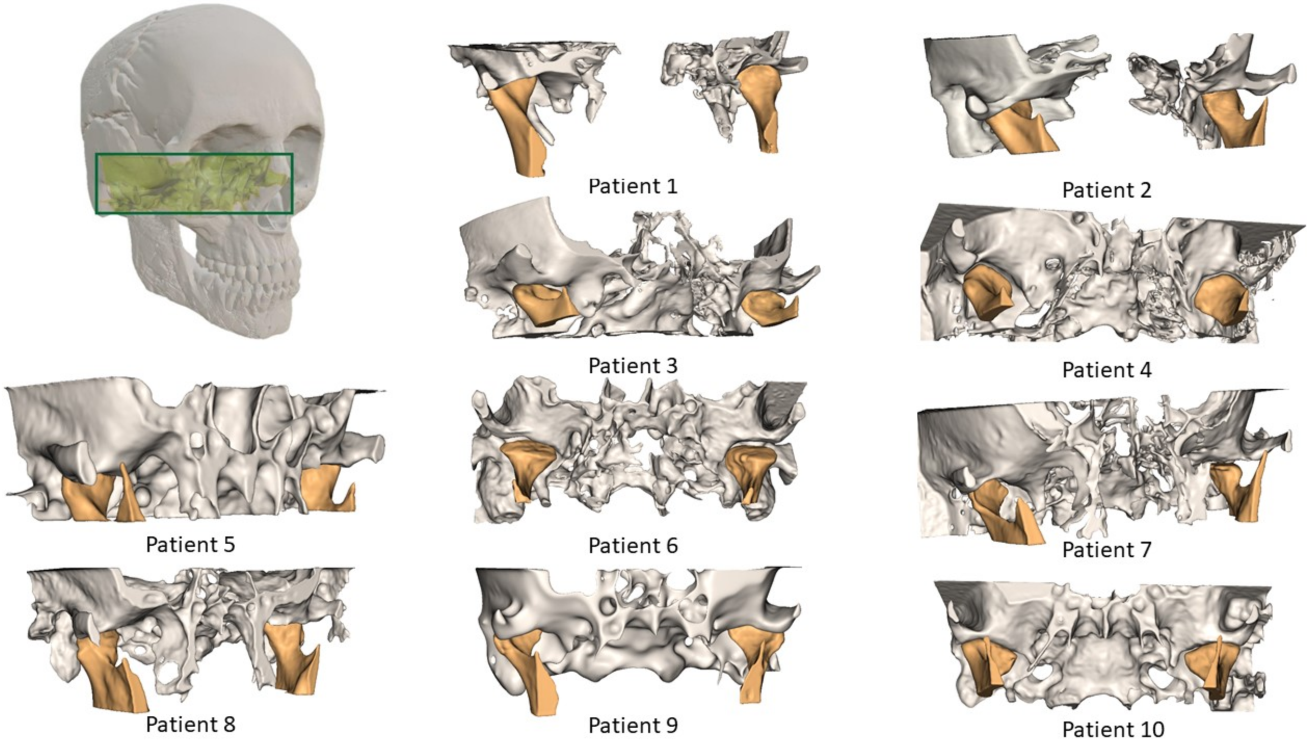

Supplementary Figure 1. Anatomical diversity of TMJ consisting of the mandibular condyle (orange) and temporal bone (gray) for 10 patients from the dataset. For 1 and 2 patients two volumes of interest (VOIs), containing left and right joint, were individually selected. While for patients 3-10 only one VOI, bounding two joints, was chosen, which results in one STL model for both temporal bones (left and right). (Figure created with Incscape v.1.1, `https://inkscape.org/`, volumes are rendered with Blender v.2.92).

2 Dataset details

To validate our deep negative volume segmentation approach, we use a local dataset containing high-resolution DICOM scans of the heads of 50 patients. The dataset was acquired at 'Clinica na Griboyedova' dental clinic (Saint-Petersburg, Russia) specially for conducting this research. There are no publicly available datasets suitable for this study because of the sensitive biometric data contained within the head CT scans (e.g., face and teeth). The authors have met the regulatory requirements necessary to submit this manuscript for publication.

Despite the fact, that all TMJ components vary considerably both in size and shape, mandibular condyle has a simple recognizable form, resembling an oval from above, while temporal bone has a much more complicated configuration due to the plenty of spikes and irregularities. Fig. S2 illustrates how complex the temporal bone structure is.

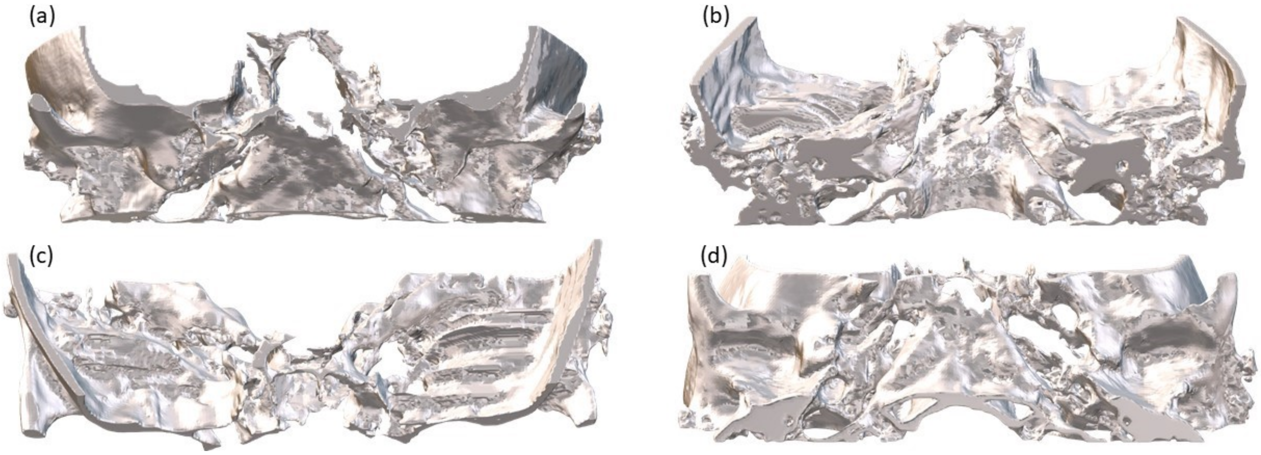

Supplementary Figure 2. One model for both temporal bone (left and right) in different views: coronal from the front (a), coronal from the back (b), axial from the top (c), axial from the bottom (d). Figure created with Incscape v.1.1, `https://inkscape.org/`, volumes are rendered with Blender v.2.92.

3 Negative Volume Segmentation

In order to extract the necessary negative volume, we first tried to implement its segmentation directly in a supervised learning manner. It proved to be a difficult task, since the outer boundaries of the negative volume represent an almost perfect sphere and are not anatomically defined. Fig. S3 shows the result of this segmentation and compare manually annotated negative volume with reconstructed one. Fig. S8 demonstrates in 2D axial slices how poorly the models try to repeat the round non-anatomical contour of the annotated "ball".

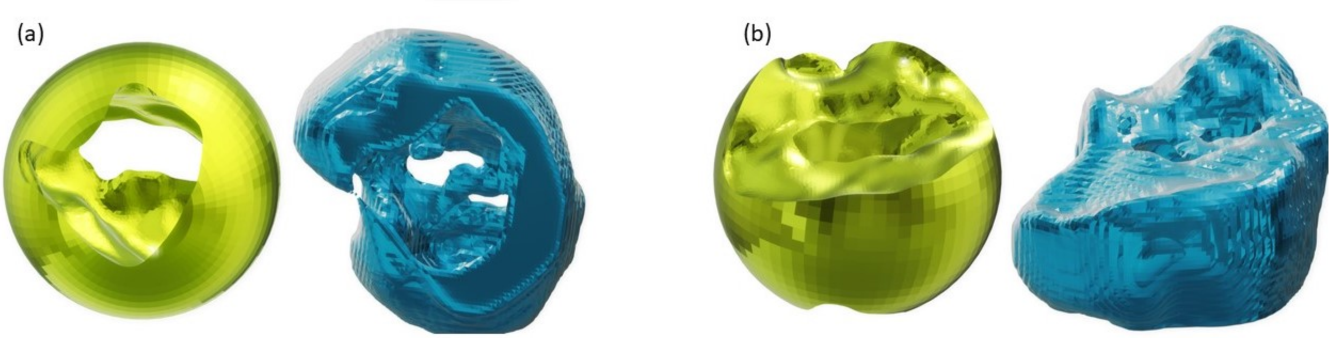

Supplementary Figure 3. Comparison of a manually annotated negative volume (green) and a reconstructed one after segmentation (blue) from the bottom (a) and from the side viewangle (b). The figure demonstrates that a straightforward segmentation of the negative volume is not capable of extracting empty space withing a spherical object well, especially if only a limited annotation is available. (Volumes are rendered with Blender v.2.92).

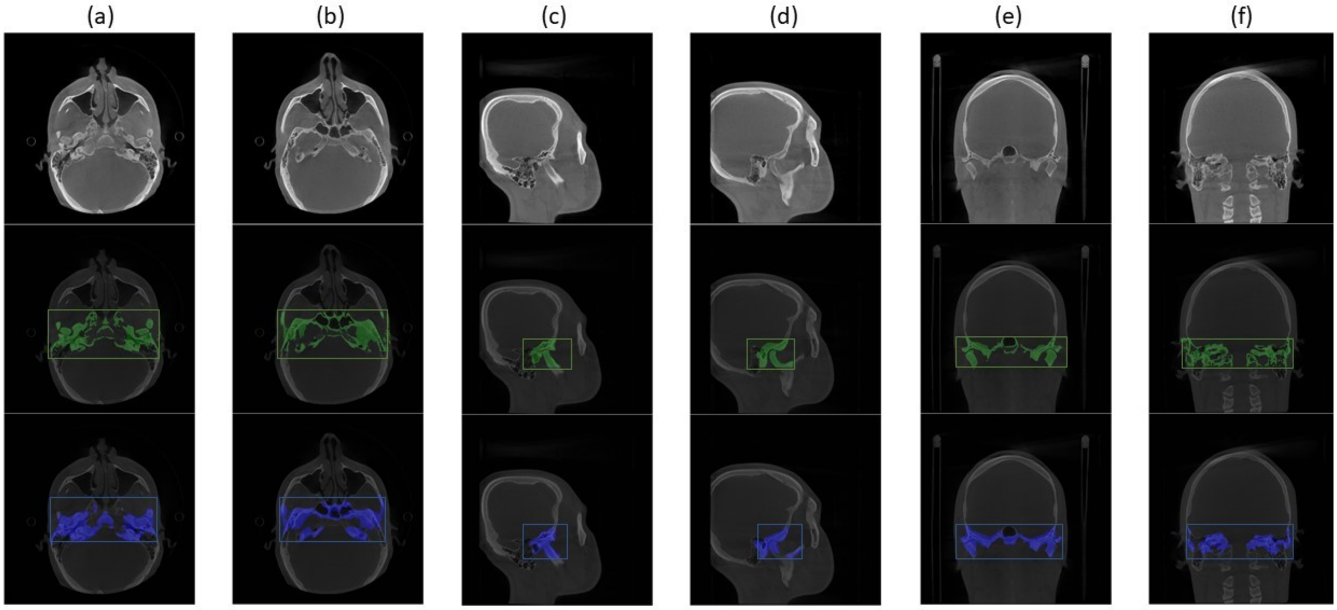

Supplementary Figure 4. Automatic VOI localization results: (a), (b) axial view; (c), (d) sagittal view; and (e), (f) coronal view. Top row: raw images. Middle row: ground truth bounding box (green). Bottom row: automatic localization output (blue). Figure created with Incscape v.1.1, `https://inkscape.org/`, images are generated with proposed Python pipeline.

4 Localization Results

Architectures based on the Convolutional Neural Networks and related to localization of compound joints i.e. such as knee[1,2] and hips[3] are of special interest for our task, since the aim is to localize junction between bones (small adjoining parts of the bones) and not the object entirely as in case of organs or tumors. These approaches aimed to automatically detect joint region utilize a coarse segmentation to solve the localization problem.

Similarly, the localization step is essential in our case of joint structures segmentation, since we have high-resolution input ($686 \times 686 \times 686$) with small area of interest and inconsistent joint annotation in the sense that the original joint VOI was chosen not strictly anatomically but intuitively.

To perform localization of joint we utilize V-Net architecture on full CT scans and treat it as a problem of coarse segmentation at a lower resolution. Fig. S4 represents visual results of localization together with the bounding VOI for both joints in different planes. In order to facilitate further segmentation, we crop resulted VOIs into 3 parts by sagittal cross sections in a such way that right and left parts contain joints, and the middle one does not. Thus, for each CT scan localization step results in two cropped volumes which are on average equal to $144 \times 150 \times 117$ but differ slightly for all patients.

5 Segmentation Performance

5.1 Influence of the training set size

We investigate how the size of training set affects bone segmentation performance. Since only 10 annotated scans were available, we evaluate the performance of models in cross-validation procedure, increasing the number of patients in training set from 1 to 9. For the size of training set from 1 to 8 we perform 5-fold cross-validation, as for the experiments in the main part, and for the training size of 10 we use leave-one-out cross-validation.

Fig. S5 demonstrates how the size of the training set affects segmentation performance. Although the performance gain from the additional training data tends to decrease, extending the dataset can stably improves results. This is especially noticeable for MC, where Dice score of segmentation reaches 92% with 9-patient training set, while for TB performance growth looks less significant and more linear. According to our observations, when segmenting such complex structures the quality of annotation is no less important than the quantity. Future work could study how these approaches perform when more data is available.

5.2 Influence of the training loss

We compare the performances of V-Net architecture training with different loss functions: Cross-Entropy (CE), Dice loss (D), and their linear combination (D+CE) to evaluate the impact of these metrics on segmentation results. Fig. S6 demonstrates

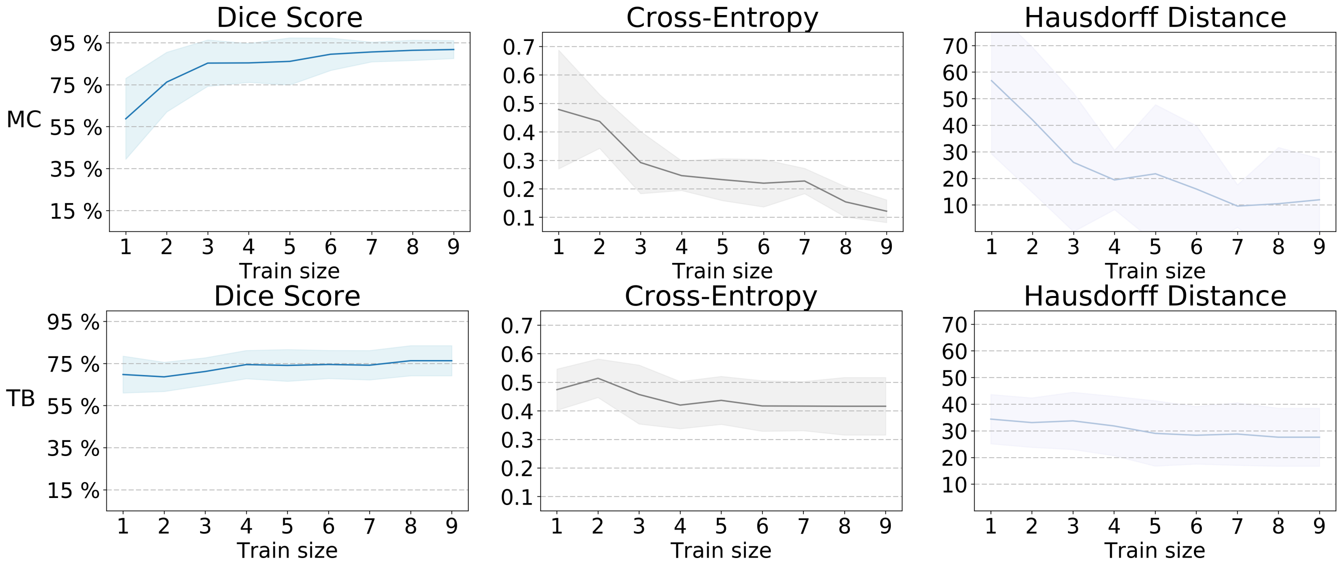

Supplementary Figure 5. Dependence of performance on the test set and on the size of the training set (the number of patients) for MC and TB segmentation in the first and the second row, respectively. Plots are generated with Matplotlib v.3.4.2.

convergence of these loss functions. For MC segmentation, the convergence rate with D+CE loss is noticeably faster for both Dice score and Cross-Entropy and resulted Cross-Entropy value is smaller. While for TB, the Dice score is almost independent of the loss selection, and with CE loss Cross-Entropy value is, as expected, slightly better. Taking into account the superiority of D+CE loss in Hausdorff distance (see Table 2 in the main part) and overall visual comparison of reconstructed bones in Fig. S11, we chose model configuration with D+CE loss for further steps.

5.3 Visual results

We provide qualitative examples to assess visually the behavior of segmentation models in 2D axial slices for MC in Fig. S9 and TB in Fig. S10, as well as in 3D reconstructed outputs for both bones in Fig. S11.

5.4 Video supplementary

The supplementary video (Video_supplementary.mp4) illustrates the proposed deep negative volume segmentation pipeline, comparing manual and fully automated approaches. The resulting negative volumes robustly and volumetrically characterize the health of a joint. Video information: standard H.264, AAC codecs; resolution 1280×720; duration 01:26; size 37.8 MB.

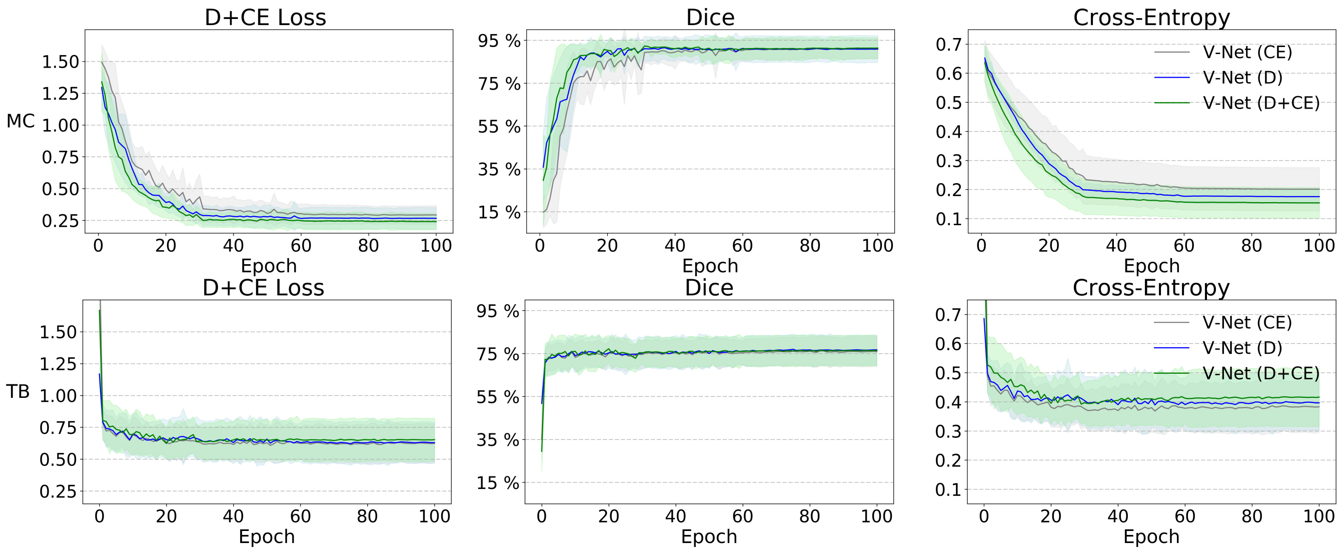

Supplementary Figure 6. Comparison of V-Net convergence on validation tests with different training loss: Cross-Entropy (CE), Dice loss (D), and their combination (D+CE). Plots are generated with the Matplotlib v.3.4.2.

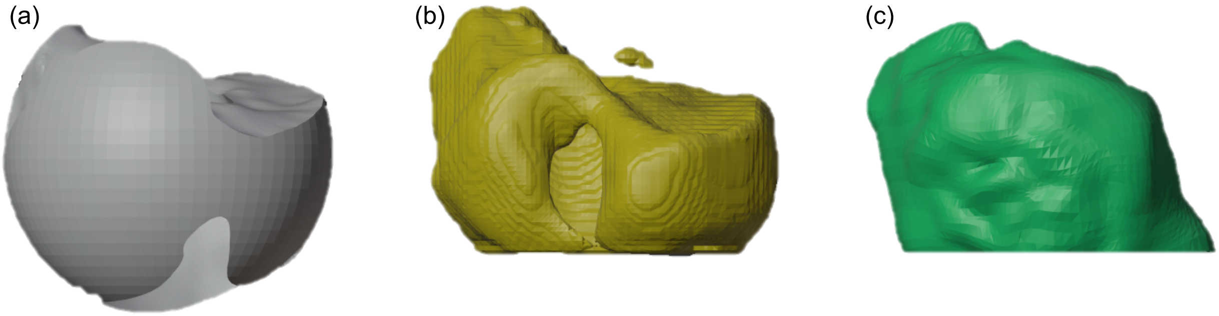

Supplementary Figure 7. Side view of the negative volumes (Patient 8) generated by: (a) manual annotation; (b) 3D segmentation given the manual annotation; (c) bone segmentation followed by the inflation procedure, as discussed in the main text. Notice that the manual annotation entails the idealistic sphere which misses important details of the true morphology within the joint. Such an idealistic annotation could be used to train a segmentation model, as shown in (b), however, the model fails to learn how to 'fill' the empty space between the bones. To the opposite, our proposed inflation method eliminates the problem and requires much less data to train the neural network (Please see the supplementary video for a better view: `Video_supplementary.mp4`). Volumes are rendered with Blender v.2.92.

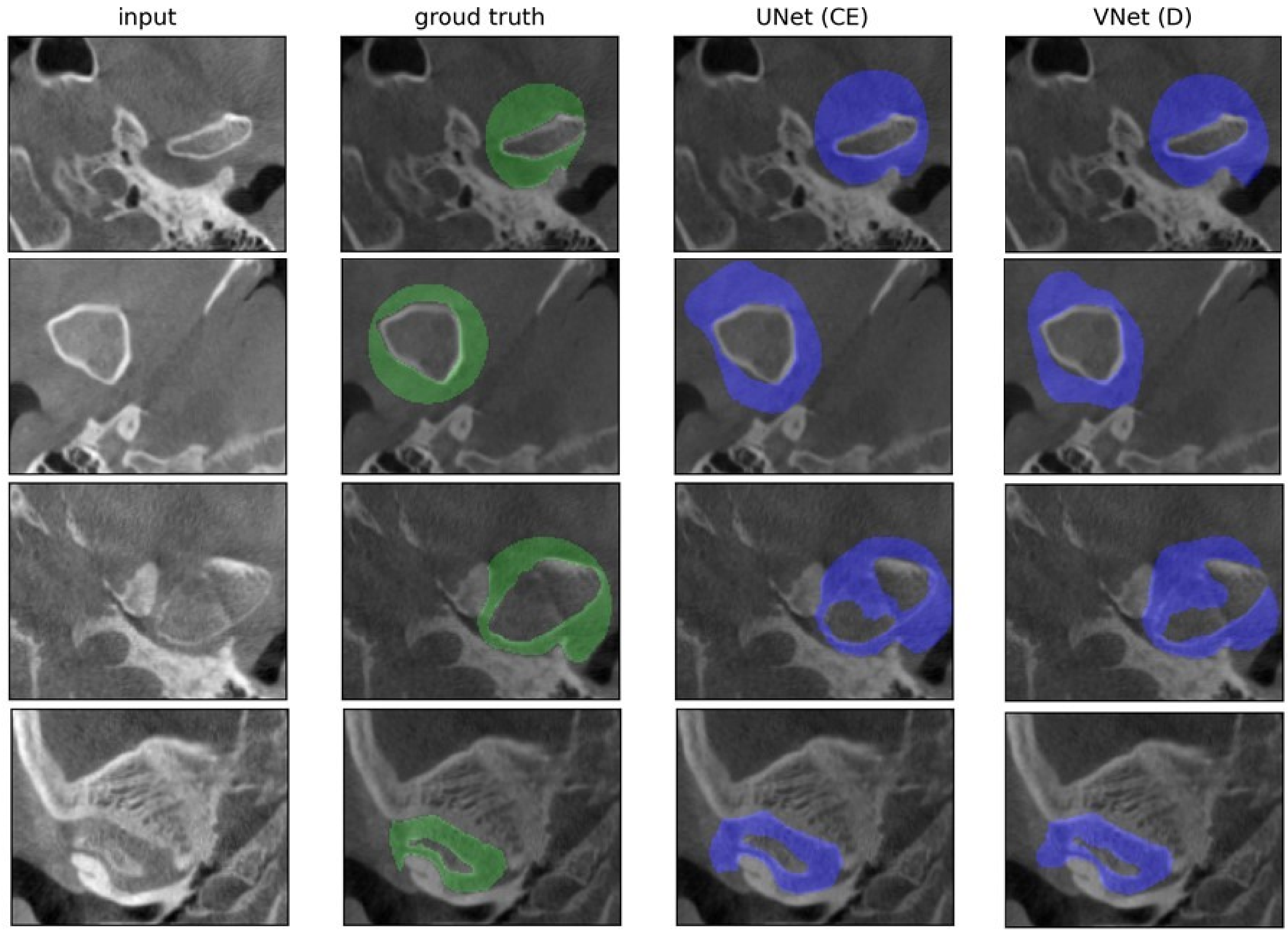

Supplementary Figure 8. Segmentation results for spherical negative volumes. Note how fruitlessly the models try to repeat the round non-anatomical contour of the "ball". Images are generated with proposed Python pipeline.

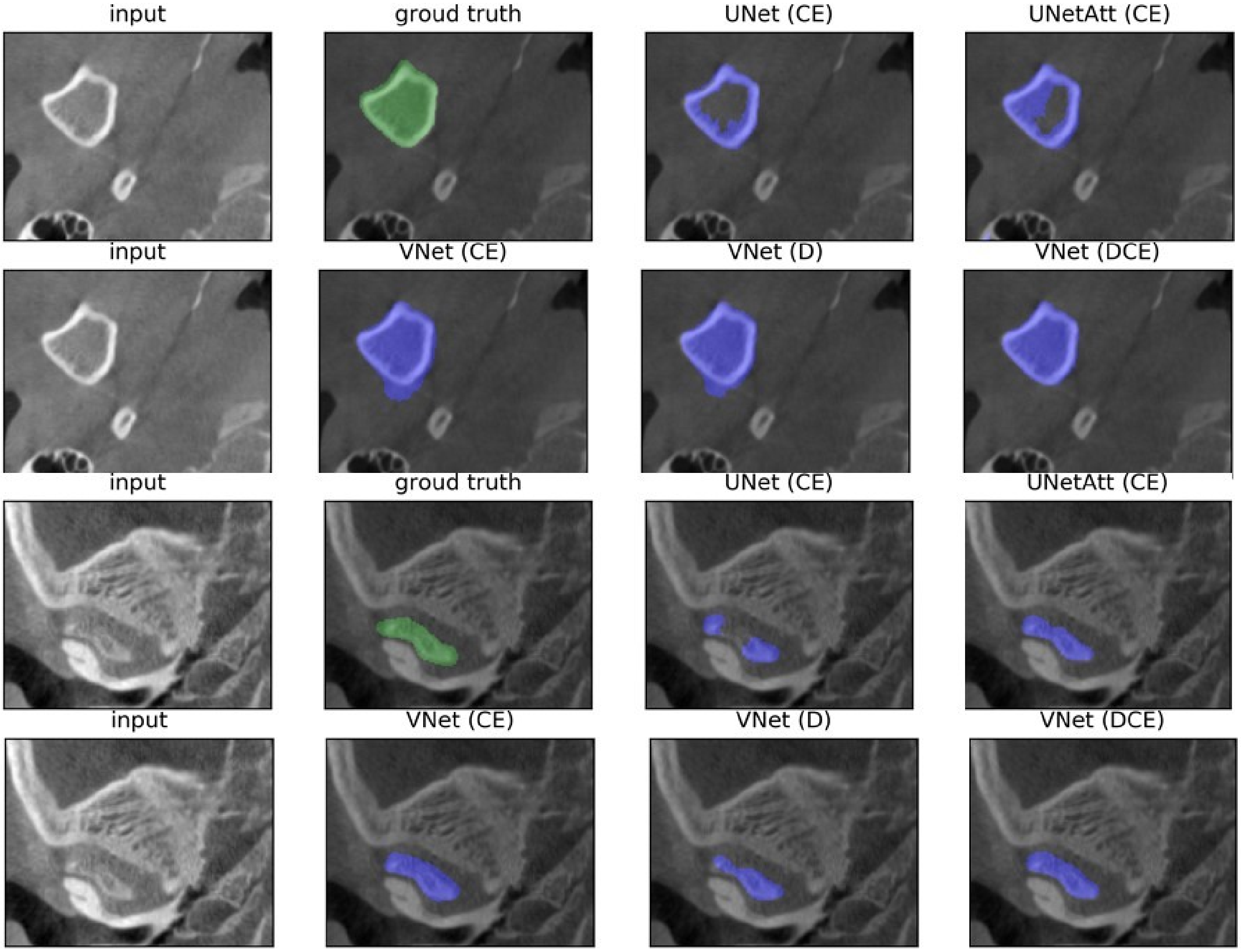

Supplementary Figure 9. Segmentation results for the most difficult examples of mandibular condyle (MC): the first input (first two rows) is different in shape from the typical examples of MC represented in the training set, and the second input (last two rows) has unclear boundary of bone tissues. Our inflation pipeline allows to mitigate and to generate the negative volumes even for these patients. Images are generated with the Python pipeline.

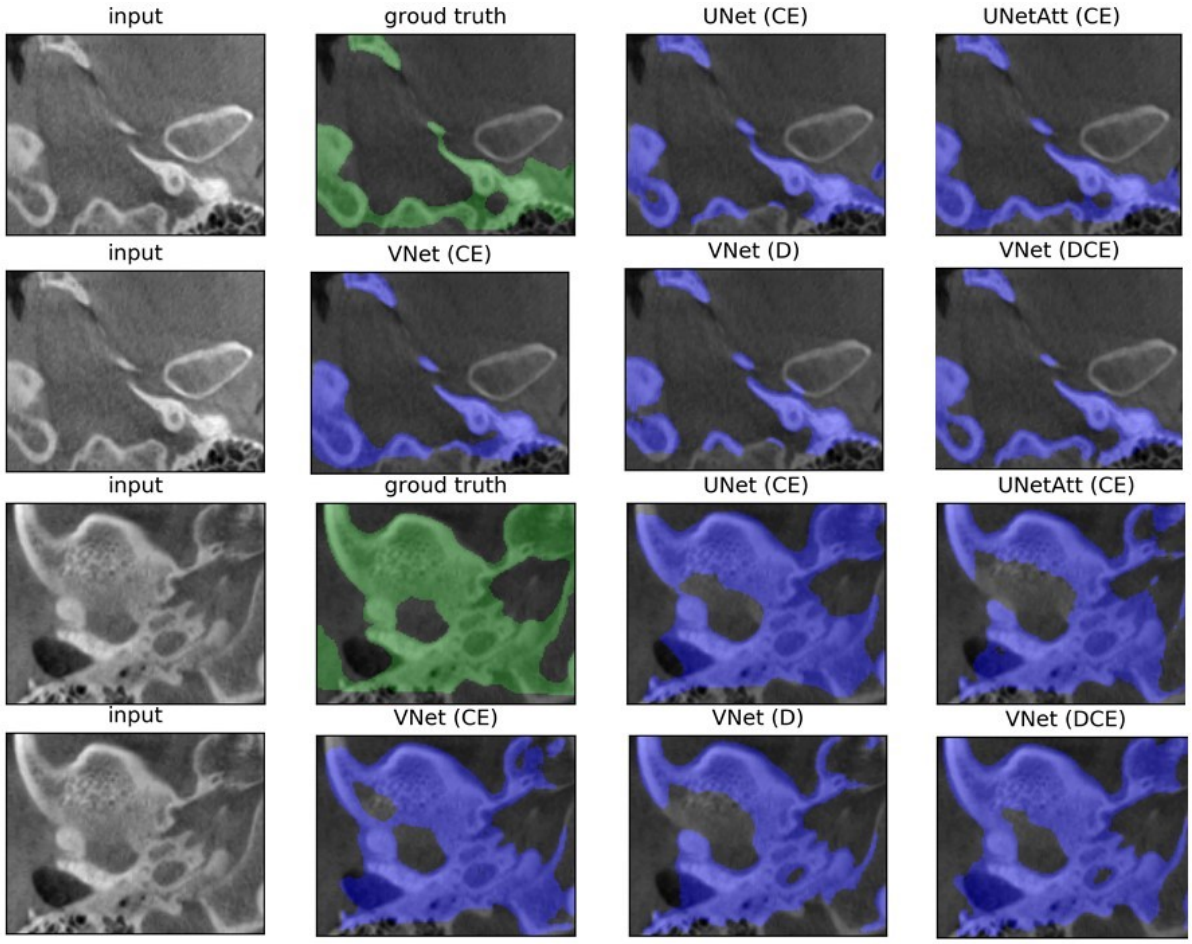

Supplementary Figure 10. Segmentation results for temporal bone. Note the fuzzy boundary of the temporal bone even in ground truth (green), that presents hard to annotate area. Images are generated with the Python pipeline.

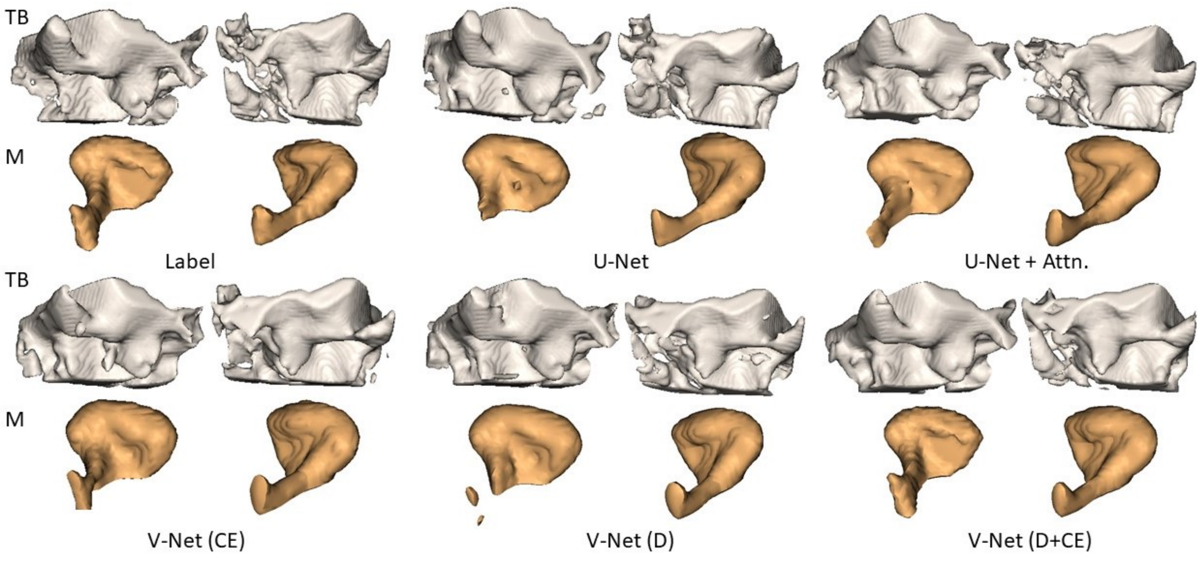

Supplementary Figure 11. Results of TMJs segmentation for a single patient using 3D U-Net, 3D U-Net with attention, and V-Net architectures. Ground truth labels and the reconstructed models for the bone components of the TMJ: temporal bone (TB, gray) and mandibular condyle (MC, orange) are shown. Images are generated with the Python pipeline, volumes are rendered with Blender v.2.92.

References

1. Sun, Y., Teo, E. C. & Zhang, Q. H. Discussions of knee joint segmentation. In *2006 International Conference on Biomedical and Pharmaceutical Engineering* (IEEE, 2006).

2. Antony, J., McGuinness, K. & O'Connor, N. E. Automatic detection of knee joints and quantification of knee osteoarthritis severity using convolutional neural networks. *Machine Learning Data Mining Pattern Recognition* DOI: 10.1007/978-3-319-62416-7_27 (2017).

3. Tang, M., Zhang, Z., Cobzas, D., Jagersand, M. & Jaremko, J. L. Segmentation-by-detection: A cascade network for volumetric medical image segmentation. In *ISBI 2018*, 1356–1359, DOI: 10.1109/ISBI.2018.8363823 (IEEE, 2018).
